# Genotype-phenotype correlation of *WT1* mutation-related nephropathy in Chinese children

**DOI:** 10.3389/fped.2023.1192021

**Published:** 2023-07-27

**Authors:** Huanru Chen, Miao Zhang, Jinai Lin, Jieyi Lu, Fazhan Zhong, Fu Zhong, Xia Gao, Xin Liao

**Affiliations:** Department of Nephrology, Guangzhou Women and Children’s Medical Center, Guangzhou Medical University, Guangzhou, China

**Keywords:** *WT1 gene*, hereditary nephropathy, end-stage renal disease, steroid-resistant nephrotic syndrome, Denys-Drash syndrome, Frasier syndrome

## Abstract

**Introduction:**

This study aimed to analyze the clinical characteristics of nephropathy associated with *WT1* gene mutations in Chinese children and explore the relationship between genotype and clinical phenotype.

**Methods:**

Cases diagnosed at the Guangzhou Women and Children's Medical Center, were combined with those retrieved from PubMed and China National Knowledge Infrastructure (CNKI) databases from January 2015 to June 2022 and integrated into a study cohort; grouped according to gene mutation sites, clinical phenotype, and renal pathological types. The clinical characteristics between groups were compared, and the relationship between genotype and age of onset, clinical phenotype, and pathological type were retrospectively analyzed.

**Results:**

The center enrolled 15 confirmed children: seven cases of non-simple nephropathy, including Denys-Drash syndrome (DDS) and Frasier syndrome (FS); eight cases of isolated steroid-resistant nephrotic syndrome (ISRNS); and 13 cases (86.7%) that progressed to end-stage renal disease (ESRD). The initial hemoglobin and bicarbonate levels of patients with clinical non-simple nephropathy were significantly lower than those with simple nephropathy, whereas the serum creatinine levels were higher than those of patients with simple nephropathy. A total of 75 cases of nephropathy associated with *WT1* mutations in the study cohort met the inclusion and exclusion criteria. The most common clinical manifestations of *WT1* mutations in this cohort were DDS (29/75, 38.7%) and ISRNS (37/75, 49.3%). A renal biopsy was performed in 43 patients, and the common types of renal pathology were focal segmental glomerulosclerosis (23/43, 53.5%) and DMS (13/43, 30.2%). Within the cohort, there were 12 cases (16.0%) in the exon 8 mutation group, 32 (42.6%) in the exon 9 group, 19 (25.3%) in the intron 9 group, and 12 (16.0%) in other gene site mutation groups. Common sites of *WT1* mutations in Chinese children were exons 9 and intron 9. Exon 8 mutations were uniquely correlated with the age of onset within three months [5/7; 71.4%; Adjusted standardized residual (AR) = 4.2]. The renal survival time in the exon 8 mutation group was the shortest (*P* = 0.003).

**Discussion:**

The molecular and biological characteristics of *WT1* mutation-related nephropathy determine the clinical type, pathological features, and renal survival time of the disease; and there was a strong correlation between the genotype and clinical phenotype.

## 1. Introduction

The Wilms' tumor gene 1 (*WT1*) is important for the normal development of the kidney and reproductive system and plays a key role in podocyte development (1, 2). *WT1* mutation causes a variety of glomerular diseases, including Denys-Drash syndrome (DDS), Frasier syndrome (FS), and isolated steroid-resistant nephrotic syndrome (ISRNS). A Chinese study of 278 cases of steroid-resistant nephrotic syndrome (SRNS) with definite gene mutations showed that the incidence of *WT1* mutations was 5.4% and that it was the second most common causative gene of SRNS (3). A study of 127 children with monogenic SRNS from South Korea showed that the incidence of *WT1* mutations was 23.6% and that it was the most common pathogenic gene in SRNS (4). In this study, we retrospectively summarized the clinical characteristics of children with *WT1* mutation-related nephropathy at the Guangzhou Women and Children's Medical Center and searched the Chinese literature on *WT1* mutation-related nephropathy to further understand the effect of these mutations. We analyzed the relationship between the genotype and clinical phenotype of *WT1* mutation-related nephropathy in Chinese children. This study provides evidence for the diagnosis, treatment, prognostic assessment, and management of *WT1* mutation-related nephropathy.

## 2. Materials and methods

### 2.1. Study design and participants

The subjects of the study were children diagnosed with *WT1* mutation-related nephropathy by genetic testing at the Nephrology Department of Guangzhou Women and Children's Medical Center between January 2015 and June 2022. The PubMed and China National Knowledge Infrastructure (CNKI) databases were searched for cases of Chinese children with *WT1* mutation-related nephropathy from 2015 to 2022.

This retrospective case series study was divided into two parts: first, the analysis of the clinical characteristics of the cases in our center; and second, the correlation analysis between the genotype and clinical phenotype of the cases in our center and the literature search cases integrated into a case cohort.

### 2.2. Collection of clinical data from cases in Guangzhou women and children's medical center

The hospital's electronic medical record system was queried to determine the general situation of the children (sex, age at onset, age at diagnosis), clinical manifestations (first symptom, treatment, complications), laboratory examinations, pathological examinations, prognosis [death, progression to end-stage renal disease (ESRD), or kidney transplantation], and other data. This study was conducted in accordance with the tenets of the Declaration of Helsinki and was approved by the Ethics Committee of Guangzhou Women and Children's Medical Center (approval number: [2022] 189A09). Informed consent was obtained from all participating subjects and their parents.

Inclusion criteria were children of 18 years or under at the time of diagnosis with nephropathy and genetic diagnosis of *WT1* pathogenic mutation, or suspected pathogenic mutation. Exclusion criteria were the presence of other highly pathogenic steroid resistance-related gene mutations and secondary glomerular diseases such as clinically associated hepatitis B virus, systemic lupus erythematosus, allergic purpura, and ANCA-related nephritis.

### 2.3. Gene detection method

The identification process was a two-step procedure involving high-throughput sequencing and validation of suspected disease-causing mutations by Sanger sequencing.

### 2.4. Genetic pathogenicity evaluation criteria, clinical classification, and experimental groupings

Pathogenicity analysis was performed according to the American Society of Medical Genetics and Genomics variation interpretation guidelines: pathogenic, probably pathogenic, uncertain, probably benign, and benign.

The clinical classifications were as follows: DDS, nephropathy with nephroblastoma or genitourinary abnormalities; FS, nephropathy with male pseudohermaphroditism or gonadal tumor; ISRNS, intrinsic steroid-resistant nephrotic syndrome; or persistent glomerular proteinuria.

Groups were divided by nephropathy, clinical type, renal pathology, and position of *WT1* mutation. The nephropathy grouping was divided into simple nephropathy or, where the nephropathy was complicated by tumors or genitourinary abnormalities, non-simple nephropathy. Clinical types were divided into the DDS, FS, and ISRNS groups. Depending on the renal pathology, groups were divided into focal segmental glomerulosclerosis (FSGS), DMS, and other groups. Depending on the position of the genetic variation in *WT1*, groups were divided into exon8, exon9, intron9, and other position variation groups.

### 2.5. Literature search and data extraction

PubMed and CNKI databases were searched for *WT1* mutations and glomerular diseases in Chinese children published between January 2015 and June 2022. Specifically; a keyword search using China, children, *WT1* mutations, Denys-Drash, Frasier, SRNS, glomerulopathy, and nephropathy was performed, and cases of nephropathy associated with *WT1* gene mutations in Chinese children who met the inclusion and exclusion criteria were identified. Clinical phenotype data, gene information, age at onset, prognosis, and other data were extracted. Duplicate cases with missing age at onset, specific genotypes, or clinical databases from the same research team were excluded.

### 2.6. Statistical analysis

Statistical analyses were performed with SPSS version 25.0 (IBM Corp., Armonk, NY, USA). Data that conformed to the normal distribution were represented by the mean (standard deviation) using the *t*-test, and data that did not conform to the normal distribution were represented by the median (P25, P75) using a nonparametric test. Kaplan–Meier survival analysis and the log-rank test were used to compare the differences in renal survival time between different mutation sites and clinical types. Fisher's exact test (*R* × *C*) was used to compare the differences in clinical and pathological types of different mutation sites of *WT1*. Cramer's *V* coefficient was used to represent the correlation strength, and the differences between groups were assessed by pairwise comparisons based on adjusted standardized residuals. A *P* value <0.05 and an adjusted standardized residual (AR) >2 were considered statistically significant. Graphs were generated using the Prism 9 software (GraphPad Software, San Diego, CA, USA).

## 3. Results

### 3.1. Clinical characteristics of the cohort of children in our center

Fifteen patients were enrolled in our center (Table 1); six biological males (40%) and nine biological females (60%). The overall median age of onset was 13.0 (6.0, 33.0) months, the median age of Wilms' tumor onset was 12.5 (9.8, 25.8) months, and the median age of renal disease onset was 14.0 (6.0, 46.0) months. The age of tumor onset was earlier than the age of renal disease onset. There were eight cases (53.3%) of simple nephropathy and seven cases (46.7%) of non-simple nephropathy. Clinical classification identified four DDS, three FS and eight ISRNS cases. The initial symptoms were edema (*n* = 8, 53.3%), proteinuria (*n* = 4, 26.7%), and Wilms' tumor (*n* = 3, 20%). Renal biopsy was performed in seven children and identified five cases of FSGS, one of membranous nephropathy (MN), and one case of diffuse proliferative glomerulonephritis (DPGN).

**Table 1:** The situation of 15 children with *WT1* gene mutation nephropathy in our center.

ID	Gender	WT1 mutation*	AGMC	Mutation type	First symptoms (months of onset)	Urogenital malformations	Tumor	Clinical type	Kidney pathology	ESRD (months of onset)	End point of follow-up (months)
1	Female	Exon 8:c.1301G>A p.R434H	Pathogenic	Missense	Edema (0.1)	–	–	ISRNS	–	0.25	Death (1)
2	Female	Exon 8:c.665G>A p.R222H	Pathogenic	Missense	Edema (0.23)	–	–	ISRNS	–	0.25	Death (1)
3	Female	Exon 8:c.703G>A p.G235S	Likely pathogenic	Missense	Edema (13)	–	–	ISRNS	–	13	Death (13.5)
4	Male	Exon 8:c.647G>A p.C216Y	Pathogenic	Missense	Wilms’ Tumor (13);proteinuria (14)	Hypospadias	Wilms’ tumor	DDS	–	17	Death (20)
5	Male	Exon 8:c.1239A>T p.H450l	Pathogenic	Missense	Edema (46)	Left cryptorchidism	–	FS	FSGS	46	Kidney transplant (55), normal (88)
6	Female	Exon 9:c.1390G>A p.D464N	Pathogenic	Missense	Wilms’ tumor (9); Edema (11)	–	Wilms’ tumor	DDS	–	14	Death (15)
7	Female	Exon 9:c.748C>T p.R250W	Pathogenic	Missense	Proteinuria (19)	–	–	ISRNS	FSGS	42	Death (55)
8	Female	Exon 9:c.722G>A p.C241Y	Pathogenic	Missense	Proteinuria (5); Wilms’ tumor (30)	–	Wilms’ tumor	DDS	FSGS	28	Death (31)
9	Male	Exon 1:c.512G>T p.G171V	Likely pathogenic	Missense	Edema (7.5)	Hypospadias	–	FS	–	8	HD (10.5)
10	Female	Exon 4:c.911C>T p.S304P	Likely pathogenic	Missense	Edema (6)	–	–	ISRNS	–	39	Kidney transplant (52), normal (67)
11	Male	Exon 7:c.1255A>G p.K419E	Likely pathogenic	Missense	Proteinuria (146)	–	–	ISRNS	FSGS	–	Partial remission (181)
12	Male	Intron 9:c.1432+5G>A	Pathogenic	Splicing	Proteinuria (28)	Hypospadias	–	FS	DPGN	37	Kidney transplant (89), normal (111)
13	Female	Intron 9:c.1432+5G>A	Pathogenic	Splicing	Edema (33)	–	–	ISRNS	FSGS	38	PD (101)
14	Male	Intron 7:c.1265-1G>A	Pathogenic	Splicing	Wilms’ tumor (12); Edema (107)	Hypospadias	Wilms’ tumor	DDS	–	107	Kidney transplant (111), normal (148)
15	Female	Exon1-10:Del	Likely pathogenic	Deletion	Edema (74)	–	–	ISRNS	MN	–	Partial remission (93)

* The transcripts for cases 3–5 and 7–8 are NM_001198551, and the remaining transcripts are NM_024426. FSGS, focal segmental glomerulosclerosis; MN, membranous nephropathy; DPGN, diffuse proliferative glomerulonephritis; PD, peritoneal dialysis; HD, hemodialysis.

**Figure 1:**
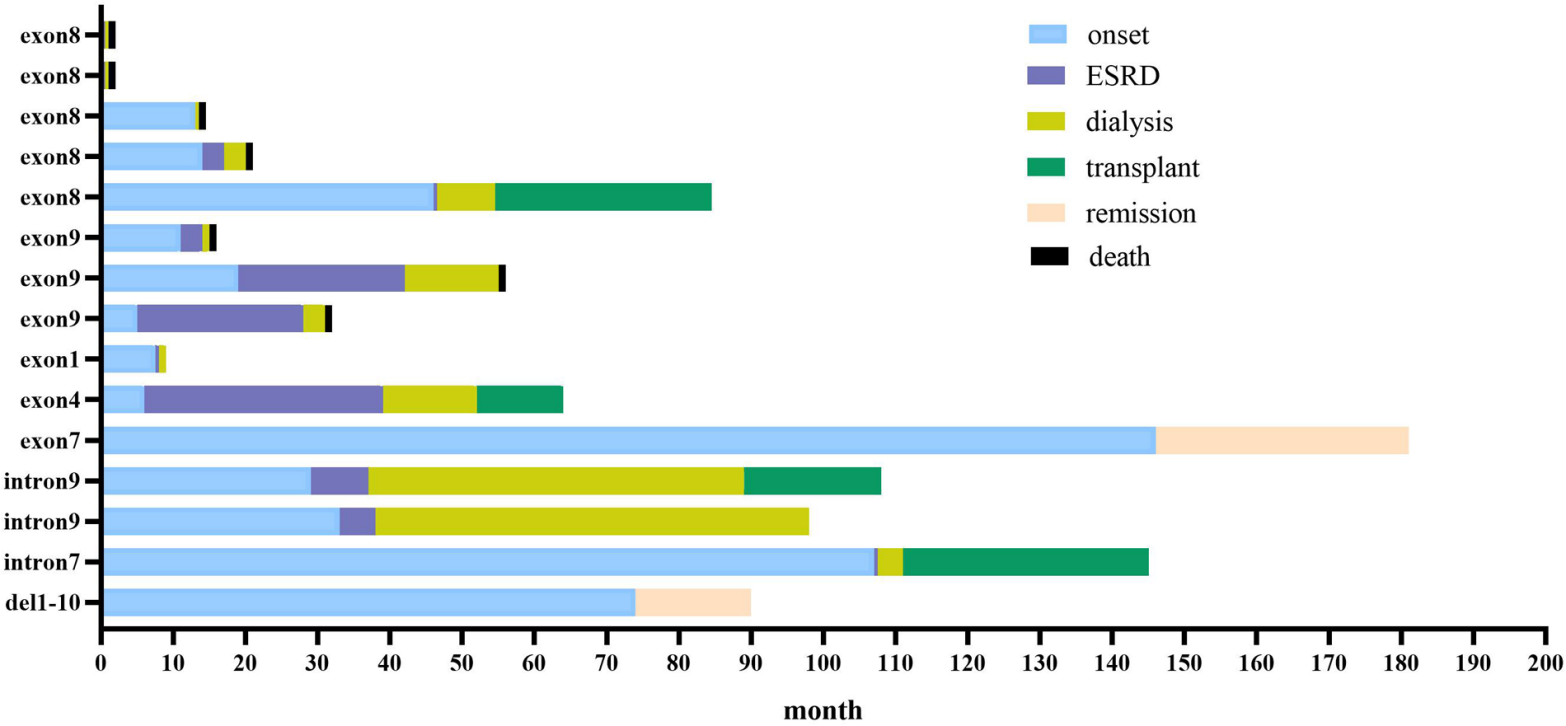
Clinical course of 15 children with WT1 mutation-associated nephropathy in our center. Blue bars show time before kidney disease; red bars show time from kidney disease to start of dialysis (kidney survival time); yellow bars show duration of dialysis; green bars show time from kidney transplantation to follow-up; orange bars show partial remission of urine protein to follow-up; black bars show death.

The median follow-up of the 15 children was 22 (3, 44) months and the longest follow-up period was 85 months. There were seven children with renal insufficiency at presentation, mean serum creatinine was 244.9 (±112.8) µmol/L; and eight children with normal renal function, mean serum creatinine was 34.4 (±12.6) µmol/L. Thirteen of the 15 children in our center progressed to ESRD (86.7%), including seven deaths, two cases of maintenance dialysis (one case each for peritoneal dialysis and hemodialysis), four cases of kidney transplantation, and two cases of partial remission of urinary protein after treatment with hormones and immunosuppressants (Figure 1).

Based on the clinical type of the enrolled children, they were divided into two groups: simple nephropathy (*n* = 8) and non-simple nephropathy (*n* = 7). Laboratory data and prognoses were compared between the two groups (Table 2). There were statistically significant differences in the initial hemoglobin and bicarbonate levels between the two groups (*P* = 0.015 and *P* = 0.033, respectively), but there were no significant differences in the urine protein to creatinine ratio (Upro/UCr), serum creatinine level, age of onset, and renal survival time (*P* > 0.05). In terms of prognosis; in the simple nephropathy group, two cases (25%) had partial remission of proteinuria, while in the non-simple nephropathy group, all cases progressed to ESRD (*n* = 7, 100%).

**Table 2:** Clinical data of simple kidney disease and non-simple kidney disease in our center.

	Simple (*n* = 8)	Non-simple (*n* = 7)	*U*/*T*/*X*^2^	*P*
Initial UPC (mg/mg)^a^	14.45 (5.46, 29.73)	21.08 (13.4, 60.3)	34.00	0.536
Initial SCr (µmol/L)^a^	44.0 (25.0, 121.5)	164.0 (31.0, 286.0)	38.00	0.281
*Initial GFR (ml/min/1.73 m^2^)^a^	114.0 (16.4, 173.4)	23.0 (14.9, 92.9)	19.0	0.336
Initial Hb (g/L)^b^	122.0 ± 18.19	99.57 ± 11.57	2.797	0.015
Initial HCO_3_^−^ (mmol/L)^a^	21.96 ± 1.65	18.14 ± 3.62	2.567	0.033
Age of onset (m)^a^	16.0 (1.69, 63.75)	12.0 (7.5, 29.0)	25.5	0.779
Kidney survival time (m)^a^	2.63 (0.19, 25.5)	3.0 (0.5, 8.0)	23.5	0.731
Remission, *n* (%)^c^	2 (25%)	0 (0%)	–	0.467
ESRD, *n* (%)^c^	6 (75%)	7 (100%)	–	0.467
Dialysis/transplant, *n* (%)^c^	2 (25%)	4 (57.1%)	–	0.315
death, *n* (%)^c^	4 (50%)	3(42.9%)	–	1.0

^a^ Median (P25, P75), nonparametric test.

^b^ *x* ± *s*, variance test.

^c^ Percentage, Fisher's exact test (2 × *C*).

^*^ Schwartz formula for calculation of glomerular filtration rate.

### 3.2. Genotype and phenotype analysis of the case cohort after integration of single center data and literature search data

#### 3.2.1. Genetic diagnostic profile of the integrated cohort

The 15 children enrolled in our center were all sporadic cases of spontaneous mutation of the *WT1* gene, and there were 14 genotypes. Four of these genotypes were reported as *de novo* mutations: case 9 (Ex1: c.512 G>T (p.G171V)), case 10 (Ex4: c.911C>T (p.S304P)), case 11 (Ex7: c.1255A>G (p.K419E)), and case 14 (Intron7: c.1265-1G>A splice). There were 11 cases of missense variation, three of splicing variation, and one deletion. Mutation sites were in exon 8 (5 cases), exon 9 (3 cases), intron 9 (2 cases), exon 1 (1 case), exon 4 (1 case), exon 7 (1 case), intron 7 (1 case), and 1 case of exon 1–0 deletion (Table 1).

A literature search of the database yielded 60 eligible cases from five individual case reports (5–9) and three cohort studies (10–12) (Table 3). A total of 75 cases from our center (*n* = 15) and retrieved literature cases (*n* = 60) were included in this research cohort, hereafter referred to as the cohort. Forty-one genotypes were identified, all of which were sporadic and nonfamilial. The most common genotypes were intron9 c.1432+5G>A splice (*n* = 10, 13.3%) and exon9 c.1384C>T (p.R462W) (*n* = 8, 10.7%).

**Table 3:** Cases of *WT1* mutation-associated glomeruli in Chinese children reported in the literature.

Author	Year	Number	Gender/Karyotype	Genotype	Clinical type	Renal pathology	Age of onset (m)	Kidney survival time (m)
Wang et al. (10)	2017	7	F	Intron8c.1339+1G>A	ISRNS	FSGS	0.2	1
			M/XY	Intron8c.1339+5G>A	DDS	FSGS	7	–
			M	Exon9c.1384C>T	DDS		9	–
			F	Intron9c.1432+1G>A	ISRNS		13	–
			F	Intron9c.1432+4C>T	ISRNS		56	18^a^
			F	Intron9c.1432+5G>A	ISRNS		28	12^a^
			F	Intron9c.1432+5G>A	ISRNS		23	15^a^
Yue et al. (11)	2017	15	F/XY	Intron9c.1228+4C>T	FS	FSGS	15	0
			F/XY	Intron9c.1228+4C>T	ISRNS	SGS	5	0
			F/XY	Intron9c.1228+4C>T	ISRNS	MCD	108	–
			F	Intron9c.1228+5G>A	ISRNS	FSGS	12	69
			F	Intron9c.1228+5G>A	FS	FSGS	21	75
			F/XX	Exon9c.1180C>T	ISRNS	DMS	60	–
			F/XX	Exon9c.1180C>T	ISRNS	MCD	0	2
			M/XY	Exon9c.1180C>T	DDS	FSGS	16	–
			M/XY	Exon9c.1180C>T	DDSDDS	DMS	25	–
			F/XX	Exon9c.1213C>G	ISRNS	DMS	22	2
			F	Exon8c.1097G>A	DDS	DMS	6	0
			M	Exon8c.1130A>T	DDS	MCD	3	22^a^
			M/XY	Exon6c.893A>T	DDS	MCD	132	–
			M/XY	Exon8c.1084C>T	DDS	DMS	132	–
			F/XX	Exon9c.1186C>T			32	–
Liang et al. (5)	2019	1	F/XY	Intron9c.1432+5G>A	FS	FSGS	84	38^a^
Sun et al. (12)	2020	33	F/XY	Exon8c.1267T>G	DDS	DMS	16	14
			M/XY	Exon8c.1301G>C	DDS	FSGS	28	18^a^
			F/XX	Exon8c.1367G>C	ISRNS	DMS	3	18^a^
			M/XY	Exon9c.1384C>T	DDS	DMS	14	36
			F/XX	Exon9c.1384C>T	ISRNS	FSGS	32.4	47^a^
			M/XY	Exon9c.1384C>T	ISRNS	FSGS	9	10
			M/XY	Exon9c.1384C>T	DDS	FSGS	19	6^a^
			F/XX	Expn9c.1384C>T	ISRNS	FSGS	15.6	56^a^
			F/XX	Exon9c.1384C>T	ISRNS	DMS	60	18^a^
			F/XY	Exon9c.1385G>A	DDS	FSGS	124.8	16^a^
			F/XX	Exon9c.1388C>T	DDS	DMS	9	6^a^
			M/XY	Exon9c.1390G>A	DDS	DMS	26.4	60
			F/XX	Exon9c.1395C>G	ISRNS	DMS	9	10^a^
			F/XX	Exon9c.1395C>G	ISRNS	FSGS	11	10^a^
			M/XY	Exon9c.1418A>G	DDS	DMS	12	36
			M/XY	Intron8c.1339+5G>A	DDS	FSGS	8	47^a^
			F/XX	Intron9c.1432+4C>T	ISRNS	FSGS	73	12^a^
			F/XX	Intron9c.1432+4C>T	ISRNS		45.6	62^a^
			F/XX	Intron9c.1432+5G>A	ISRNS		9	34
			F/XX	Intron9c.1432+5G>A	ISRNS		156	12^a^
			F/XY	Exon3c.799delG	DDS		11	6
			F/XX	Exon9c.1358G>A	DDS		6	25
			F/XX	Exon9c.1372C>T	ISRNS		72	2
			M/XY	Exon9c.1372C>T	DDS		60	65
			F/XX	Exon9c.1384C>T	ISRNS		36	3
			M/XY	Exon9c.1384C>T	DDS		12	1
			F/XX	Exon9c.1384C>T	DDS		58.8	59
			F/XY	Exonc.1390G>A	FS		15	12
			F/XY	Intron8c.1339+1G>A	DDS		8	11
			F/XX	Intron8c.1339+5G>A	ISRNS		14	1
			F/XX	Intron9c.1432+5G>A	ISRNS		12	5
			F/XY	Intron9c.1432+5G>A	FS		7	51
			F/XY	Intron9c.1432+5G>A	FS		38.4	6
Zhang et al. (6)	2020	1	F	Exon8c.1301G>A	ISRNS		0	0.23
Wang et al. (7)	2021	1	F/XY	Exon9c.1420C>T	DDS		23	46^a^
Zhang et al. (9)	2022	1	F/XX	Exon9c.1405G>A	ISRNS	FSGS	17	4
Li et al. (8)	2022	1	F/XX	Exon9c.748C>T	ISRNS	PSG	96	24

DDS, Denys-Drash syndrome; FS, Frasier syndrome; ISRNS, isolated steroid-resistant nephrotic syndrome; FSGS, focal segmental glomerulosclerosis; DMS, diffuse mesangial sclerosis; PSG, proliferative sclerosing glomerulonephritis; F, female; M, male.

^a^ Indicates no progression to ESRD at the end of follow-up.

The 75 children in this integrated cohort were divided into four groups based on the mutation sites of *WT1*: exon 8, exon 9, intron 9, and other positional mutations. There were 12 cases (12/75, 16.0%) in exon 8, 32 (32/75, 42.6%) in exon 9, 19 (19/75, 25.3%) in intron 9, and 12 (12/75, 16.0%) in the other positional mutations group. The most common locations of *WT1* mutations in Chinese children were exon 9 and intron 9.

A total of 49 cases in this cohort underwent karyotype analysis and 12 cases of sex phenotype inversion (female/XY) were found. Among them, there was one case (8.3%) of sexual phenotype inversion in the exon 8 mutation group and one case (8.3%) in the exon 9 mutation group. There were two cases (16.7%) in the intron 9 mutation group, six cases (50%) in the intron 9 mutation group, and two cases (16.7%) in the other positional mutations group.

#### 3.2.2. Kidney survival

Among the 75 children in this cohort, 42 patients progressed to ESRD, with a median age of onset of 29.0 (14.75, 48.5) months, and the median renal survival time (the time from the onset of renal disease to the development of ESRD) was 5.5 (0.88, 27.0) months.

**Figure 2:**
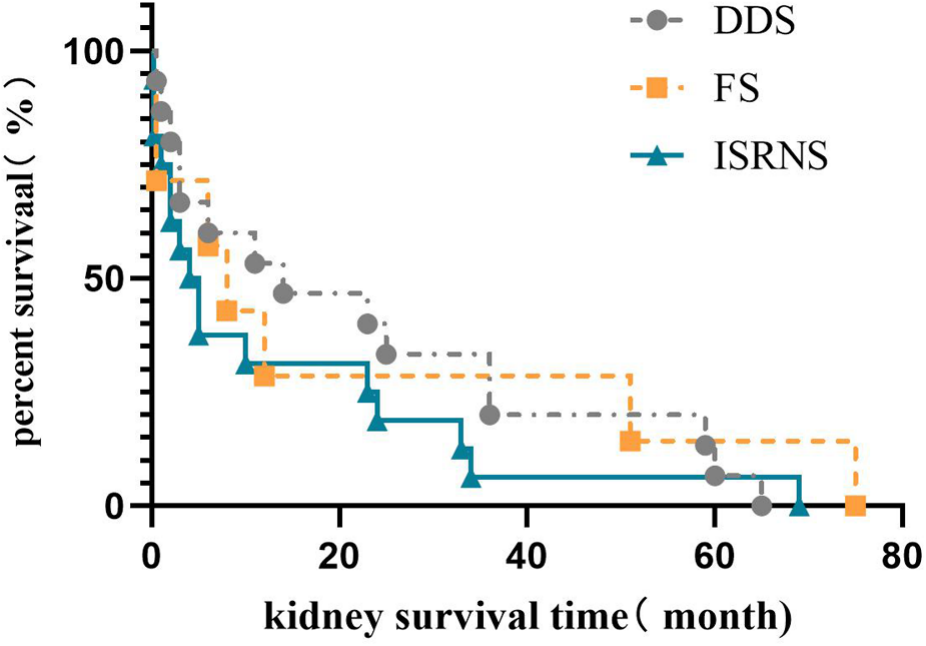
Renal survival analysis of different clinical types in Chinese children with *WT1* mutation-associated nephropathy.

According to the clinical classification, among the 42 children who progressed to ESRD, 15 (35.7%) had DDS, 8 (19%) had FS, and 19 (45.2%) had ISRNS. The median renal survival time was 14.0 (3.0, 36.0) months in the DDS group, 7.0 (0.5, 41.3) months in the FS group, and 3.0 (0.3, 23.0) months in the ISRNS group. Kaplan–Meier survival analysis showed that the renal survival time of the ISRNS group was significantly different from that of the DDS group (*P* = 0.027), and there was no significant difference in the renal survival time of the other groups (*P* > 0.05), as shown in Figure 2.

**Figure 3:**
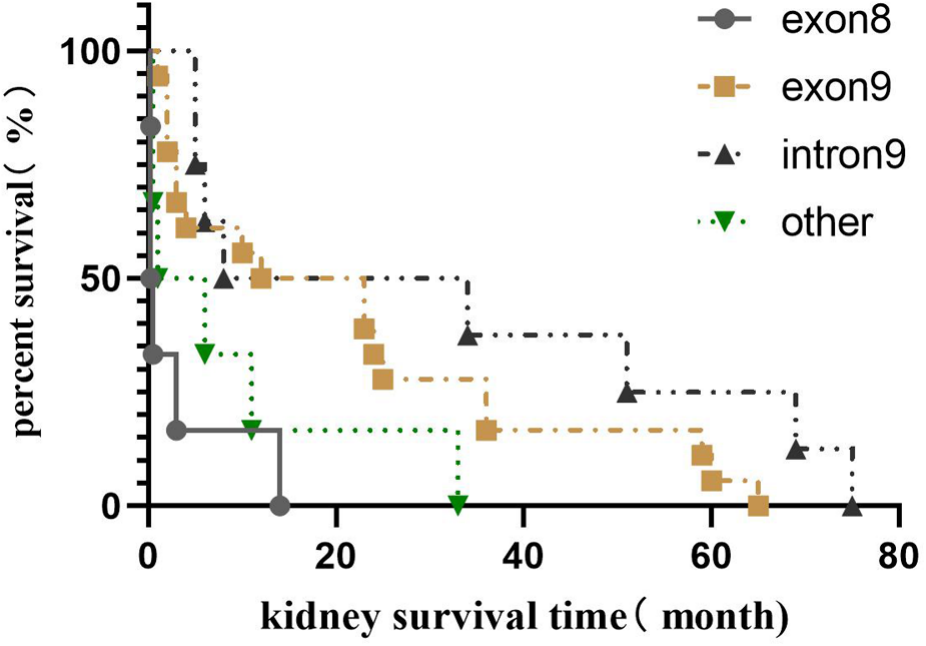
Renal survival analysis of different gene mutation positions in Chinese children with *WT1* mutation-associated nephropathy.

Among the 42 children who progressed to ESRD, 8 cases (19.0%) were in the exon 8 group, 18 cases (42.9%) in the exon 9 group, 10 cases (23.8%) in the intron 9 group, and six cases in other positional mutations group (14.3%). The median survival time of the kidneys was 0.25 (0.05, 2.38) months in the exon 8 group, 17.5 (2.8, 36.0) months in the exon 9 group, 7.0 (3.8, 55.5) months in the intron 9 group, and 3.5 (0.5, 16.5) months in the other positional mutations group. Kaplan–Meier survival analysis showed that the renal survival time of the exon 8 group was significantly shorter than that of the exon 9 and intron 9 groups (*P* = 0.000 and *P* = 0.015, respectively). There was no significant difference in renal survival time among the other groups (*P* > 0.05), as shown in Figure 3.

#### 3.2.3. Correlation analysis between *WT1* mutation locations and age at onset, clinical phenotype, and pathology

Overall, the median age of onset in the 75 children was 16.0 (9.0, 45.6) months,. The median age of onset and in the exon 8 group was 9.5 (0.9, 25.0) months; in the exon 9 group, 18.0 (11.0, 35.1) months; in the intron 9 group, 28 (12, 56.0) months; and in the other positional mutations group, 9.5 (7.1, 98.8) months. Kruskal–Wallis analysis showed that there were no significant differences in the age of onset among the four groups (*H* = 5.843, *P *= 0.119).

**Figure 4:**
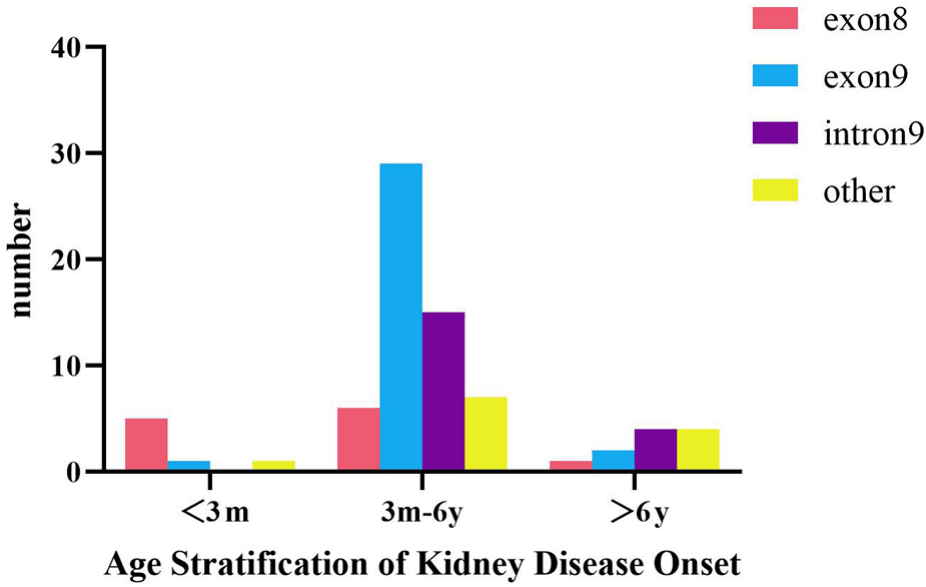
Incidence of *WT1* mutation-associated nephropathy at different ages in Chinese children.

According to age stratification (Figure 4), the age of onset among children with *WT1* mutation-associated nephropathy in China was most frequently between 3 months and 6 years of age (57/75, 76%). Those children with onset within 3 months (*n* = 7) had exon 8 mutations (5/7, 71.4%) and exon 9 mutations (29/57, 50.9%) between 3 months and 6 years of age (*n* = 57). Fisher's exact test (*R* × *C*) suggested that there was a statistically significant difference in the *WT1* mutation locations in the different age at onset groups (*P* = 0.001), and that there was a moderate correlation between the two (Cramer's *V* = 0.402; *P* = 0. 001). The results of the adjusted standardized residuals for the differences between the groups are shown in Table 4, which shows that the onset below 3 months and the exon 8 variation, and the onset between 3 months and 6 years and the exon 9 variation were statistically significant (AR = 4.2, AR = 2.6).

**Table 4:** Correlation analysis of WT1 variant sites and age of onset, clinical phenotype and pathological type in Chinese children.

	Group	WT1 mutation site			
		Exon 8	Exon 9	Intron 9	Other
Age of onset	<3 months	5 (4.2)	1 (−1.6)	0 (−1.6)	1 (−0.1)
	3 months–6 year	6 (−2.3)	29 (2.6)	15 (0.3)	7 (−1.6)
	>6 years	1 (−0.7)	2 (−1.8)	4 (0.9)	4 (2.0)
Clinical type	DDS	5 (0.2)	18 (2.7)	0 (−4.0)	6 (0.9)
	FS	1 (−0.4)	1 (−2.0)	6 (3.0)	1 (−0.4)
	ISRNS	6 (0.1)	13 (−1.3)	13 (1.9)	5 (−0.5)
Pathological	FSGS	1 (−1.6)	7 (−1.0)	13 (2.8)	2 (−1.1)
	DMS	3 (1.5)	7 (1.5)	1 (−2.6)	2 (0.2)
	Other	1 (0.2)	2 (−0.5)	2 (−0.5)	2 (1.2)

The adjusted standard residuals (AR) are in the brackets, and AR > 2 indicates that the difference is statistically significant.

The clinical classification of the 75 children showed that 29 (38.7%) had DDS, 9 (12.0%) had FS, and 37 (49.3%) had ISRNS. Fisher's exact test (*R* × *C*) suggested that there were significant differences in clinical types caused by different mutation sites in *WT1* (*P* = 0.000). The *WT1* mutation locations showed a moderate correlation with the clinical type (Cramer's *V* = 0.372; *P* = 0.002). The adjusted standardized residuals for the differences between the groups are shown in Table 4. The correlations between DDS and exon 9 mutants and between FS and intron 9 mutants were statistically significant (AR = 2.7; AR = 3.0).

Forty-three of the 75 children underwent renal pathological biopsy, including 23 (53.5%) with FSGS, 13 with DMS (30.2%), 4 with MCD (9.3%), 1 with PSGN (2.3%), one case of MN (2.3%) and one case of DPGN (2.3%). The 43 cases with renal pathological results were divided into three groups, FSGS, DMS, and the other three types; and Fisher's exact test (*R* × *C*) showed that the different pathological types caused by different mutation sites of *WT1* were statistically significant (*P* = 0.04). There was a moderate correlation between the *WT1* mutation sites and renal pathological types (Cramer's *V* = 0.357, *P* = 0.085). The results of the adjusted standardized residuals for the differences between the groups are shown in Table 4. The FSGS and intron 9 variations were statistically significant (AR = 2.8).

## 4. Discussion

*WT1* is located on chromosome 11p13 and comprises 10 exons. Exons 1–6 encode the transcriptional regulatory region and exons 7–10 encode four C2H2 zinc-finger structures that together form a zinc-finger protein with a specific DNA-binding domain (1). The WT1 protein is closely involved in the regulation of nephron formation in the embryo and the maintenance of glomerular filtration function after birth (13, 14). Experimental studies have shown that *WT1* knockout in adult mouse podocytes results in proteinuria, foot process loss, and glomerulosclerosis (15).

To date, this study contains the largest case cohort of children with *WT1* mutant nephropathy in China. It summarizes the disease characteristics and analyzes the genotype and clinical phenotypes from a new perspective using different mutation sites as subgroups.

In this cohort the age of onset of nephropathy was 16.0 (9.0, 45.6) months, which was similar to the age of onset of *WT1* mutation related nephropathy in Europe and the Middle East (Podonet Alliance) of 14.4 (3.6–51.6) months. Forty-two (56%) cases developed ESRD, with a median age of onset of ESRD of 29.0 (14.75, 48.5) months. This was later than that of the 39 cases (74%) who developed ESRD in the Podonet Alliance cohort (*n* = 53) who started continuous renal replacement therapy (RRT), with a median age of 20.4 (7.2, 93.6) months (16). In addition, we found that the *WT1* nephropathy mutation hotspots in Chinese children were located in exon 9 and intron9, which were different from those in Europe and the Middle East, which are located in exons 8 and 9 (16).

Lehnhardt et al. showed that exon 8 or 9 missense mutations had an earlier onset of kidney disease and progress to ESRD faster than intron 9 KTS splice mutants (17). In our study, children with an onset within three months of age were mainly found to have exon 8 mutations. In addition, children with exon 8 mutations had significantly shorter renal survival times than those with exon 9 or intron 9 mutations (*P* < 0.001 and *P* = 0.003, respectively) and progressed to ESRD faster. This shows that the exon 8 mutation is associated with an earlier onset of nephropathy and faster progression of the disease. Nagano et al. showed that this mutation reduces the transcriptional activity of *WT1* and affects the function of WT1. The severity of the mutation can be assessed by measuring the transcriptional activity of *WT1* (18). We speculate that the exon 8 mutation significantly reduces the transcriptional activity of *WT1* compared with mutations in exon 9 and intron 9. In addition, the C2H2 zinc finger structure is stabilized by the coordination of zinc atoms between two conserved cysteine residues at one end of the β-sheet and two conserved histidine residues at the C-terminus of the α-helix (19). Cysteine-histidine is conserved and forms the hydrophobic core of the α-helix (20). Mutations in the coding C2H2 region can cause changes in the volume and hydrophobicity of amino acids in zinc-finger proteins, resulting in changes in protein conformation and disruption of the structural stability; leading to WT1 protein dysfunction (18). Exons 8 and 9 encode zinc finger 2 and 3 structures, respectively. Our study found that exon 8 mutations were associated with a higher disease severity than those in exon 9. We postulate that mutants of different zinc-finger structures in the DNA-binding domain have different effects on zinc-finger protein stability; leading to different degrees of WT1 dysfunction. Future studies should analyze protein function to confirm this theory.

The main pathological types of *WT1* mutation-related nephropathy in Chinese children were FSGS (23/43, 53.5%) and DMS (13/43, 30.2%), which is consistent with the results reported by Auber et al. (21). Abnormalities of exons 8 and 9 in the DNA-binding domain manifest as DMS, and the intron 9 KTS splicing mutations are often associated with FSGS. In terms of clinical type, the mutations in exon 9 manifested as DDS (18/32, 56.3%) and intron 9 splicing mutations manifested as ISRNS (13/18, 72.2%). The possible presentation of isolated steroid-resistant nephrotic syndrome is consistent with the findings of FSGS on biopsy (16).

The six cases of FS in this cohort were all intron 9 mutations and all had female/XY sex phenotype inversion. We confirmed that intron 9 highly correlated with FS (AR = 3.0). Exon 9 of *WT1* encodes three amino acids, lysine, glutamic acid, and serine (KTS), which are located between the third and fourth zinc finger structures and produces two protein subtypes: +KTS and −KTS. In humans, +KTS/−KTS is normally expressed at a constant ratio of approximately 2:1 (13). Studies have shown that intron 9 mutations affect the splicing of KTS, leading to a decrease in the +KTS type and +KTS/−KTS ratio, resulting in abnormal gonadal development and sex phenotype inversion (22). Therefore, intron 9 mutations are associated with a high risk of glandular tumors. Children with genetically diagnosed intron 9 mutations should undergo karyotype analysis and early surveillance for gonadal tumors. However, intron 9 mutations appear to prevent the occurrence of Wilms’ tumors. There have been no previous reports of children with KTS splicing mutations in intron 9 developing Wilms’ tumor, and none of the 19 children with intron 9 mutations in this cohort developed Wilms' tumor. Auber also suggested that Wilms' tumors occur with any type of *WT1* mutation except those in intron 9 (21, 23, 24), although the reason for this is unclear. However, it is worth noting that the mutation of intron 9 only changes the balance of the ratio of +KTS/−KTS isoforms, rather than changing the amino acid sequence of the WT1 protein (25), therefore, the splicing variation of intron 9 will cause the +KTS isoform to be lost and not produce aberrant WT1 protein. We postulate that the +KTS isoform may be a driver of a signaling pathway that activates Wilms' tumor proliferation.

The analysis of clinical data from the 15 children in our center showed that the hemoglobin and bicarbonate ion levels were 99.57 (±11.57) g/L and 18.14 (±3.62) mmol/L, respectively, at the onset of nephropathy in children with tumors/urogenital malformations. compared with children with pure kidney disease (*P* = 0.015, *P* = 0.033). We found that children with tumors or urogenital malformations were in a state of mild anemia and metabolic acidosis at the onset of kidney disease. This indicates that the ability of the kidneys to produce erythropoietin (EPO) and regulate the acid hydrolysis balance is reduced, and renal function is impaired in the early stages of non-simple renal disease in children. The initial glomerular filtration rate of children with tumors or urogenital malformations was 23.0 (14.9, 92.9) ml/min/1.73 m^2^, which was significantly lower than that of children with pure kidney disease 114.0 (16.4, 173.4) ml/min/1.73 m^2^, however, the difference was not statistically significant (*P* = 0.336) and a larger sample size may need to be collected for analysis. Previous studies found that the WT1(−KTS) subtype, as a transcription factor, is an effective activator of EPO gene expression (26) and that the mutant WT1(−KTS) protein affects EPO gene activity. Therefore, the abnormal proliferation of combined tumors consumes nutritional energy in the body, which may aggravate the degree of anemia.

The best treatment for hereditary nephropathy is kidney transplantation, and studies have shown that recurrence of kidney disease after kidney transplantation for hereditary nephropathy is very rare (27). Roca et al. found no recurrence in five children with *WT1* mutation-related nephropathy who underwent kidney transplantation after an average follow-up of 16 years (28). The prognosis of the four children who underwent kidney transplantation in our center was also good, and no abnormalities were found during the median follow-up period of 2.1 years. The use of hormones and immunosuppressants for hereditary SRNS remains controversial. Arroyo-Parejo et al. suggested avoiding the use of immunosuppressive agents in the treatment of *WT1* nephropathy (29); however, Yue et al. found that the use of calcineurin inhibitors was effective in patients with missense and nonsense mutants (11). In our center, cases 11 and 15 were steroid resistant, and their pathologies included FSGS and stage III membranous nephropathy, respectively. The urine protein levels were partially alleviated by subsequent cyclophosphamide and tacrolimus treatment. The kidneys of these two children are still viable after three years of follow-up, and their clinical specificity may be related to the pathogenicity of the mutated gene and the functional expression of the gene-encoded protein.

*WT1* is expressed in podocytes throughout life and is critical for the functional integrity of the glomerular filtration barrier, therefore, nephropathy associated with *WT1* mutations is dominated by podocyte damage. Stem cell therapy is an innovative strategy for treating podocyte damage. Mesenchymal stem cell supplementation in azithromycin-induced rat nephropathy models maintains podocyte viability, reduces glomerular inflammation, and delay the progression of glomerular sclerosis (30). Stem cell therapy for podocyte-damaged nephropathy still requires further research to progress from experimental models to clinical application (31). In addition, recent studies have shown that knock out the of the sphingoline kinase 2 (*Sphk2*) gene or using the highly selective Sphk2 inhibitor, SLM6031434, can upregulate *WT1* expression in podocytes, protect against podocyte disease, and prevent proteinuria (32). Therefore, *Sphk2* may be a potential new target for the treatment of *WT1* mutation-related nephropathy.

In conclusion, the molecular biological characteristics of *WT1* gene mutation-related nephropathy determine the clinical type, pathological characteristics, and renal survival time of the disease, and there is a high correlation between genotype and clinical phenotype. *WT1* mutation hotspots in exon 9 or intron 9 were the main factors, and exon 8 mutations were more common in onset within 3 months of age, whereas exon 9 mutations were more common in preschool onset. Compared with exon 9 and intron 9, patients with mutations in exon 8 mutations had the shortest kidney survival time. Future research should utilize a larger cohort with longer follow-up times and conduct comprehensive analyses to establish a scientific management plan for *WT1* mutation-related nephropathy.

## Data Availability

The original contributions presented in the study are included in the article, further inquiries can be directed to the corresponding author.
